# Westminster Hospital

**Published:** 1832-04

**Authors:** 


### WESTMINSTER HOSPITAL.

### *Tetanus from a Puncture of the Foot: Death.**

Henry Roberts, a fine, well-grown lad, twelve years old, was admitted on the 7th December, with a spasmodic affection involving nearly the whole muscular system. About ten days previous to admission, the boy had his foot pierced by a rusty nail, in the sole, near the first joint of the great-toe. Three days after the occurrence of the accident, he began to experience involuntary contrac-

* The Lancet.

tions in the nether limbs; these gradually extended, and increased in severity, until the case assumed the unequivocal character of tetanus. He remained in this state with his parents nearly a week, having little or no medical assistance. When brought to the hospital, the boy presented a remarkable appearance; all the muscles of expression were contracted to the utmost, giving to the countenance a peculiarly painful character; the eyes were drawn back into the orbit; the muscles of the jaw, neck, back, sides, and limbs, were perfectly rigid, and every four or five seconds a spasm would occur, bending the body of the patient into the form of an arch, the extreme points being the head and heels; every fifth or sixth spasm greatly exceeded the rest in force, and gave the most acute pain. The boy's intelligence was unimpaired, and he could speak intelligibly notwithstanding the trismus. The action of the heart and arteries was irregular; the bowels had been brought to act, but not freely. The strength was evidently much exhausted, for nothing but pultaceous food could be given, and Mr. Lynn, therefore, thought it inadmissible to have recourse to any powerful agents. Aperient clysters were freely used, and large doses of opium were administered. Occasional intervals of sleep, but of short duration, were thus procured, and at these periods the muscles became relaxed; but the excitement of the slightest movement or sound roused his morbid sensibility, and aggravated the convulsions. These intervals of rest were insufficient to restore the system from the excessive fatigue of the tetanic movements, and the vital power was manifestly sinking. He continued in this state for about twenty-four hours, and expired quietly.

*Sectio cadaveris.* Immediately after death, the body was placed prone, to prevent the gravitation of the blood towards the spine. Twenty hours after the demise of the patient, he was laid on the dissecting table, and his spine was opened. There was slight sanguineous extravasation between the bony sides of the vertebral canal and the theca vertebralis. On opening the theca, a serous effusion was discovered, so copious in the sacral part of the spine as to produce compression. There was also a considerable quantity of serum in the cervical portion of the theca; the spinous arteries and their filaments were injected. In the cranium, the brain and its meninges were more than naturally distended with blood, and in each lateral ventricle half an ounce of a pink-coloured serum was found; the velum interpositum and choroid plexus were also unusually turgid. There was no evidence of disease in any of the other viscera.

---

### *Wound of the Orbit by a Tobacco-pipe; Penetration into the Cavernous Sinus: Death.**

Michael Walsh, an Irish lad, fifteen years old, and employed as a bricklayer's labourer at Mr. Guthrie's Eye Dispensary, in Chandos street, quarrelled, in the beginning of January last, with

* Ibid.

one of his countrymen, whilst sitting at the same table, in a public-house. During the heat of the argument, his opponent, who sat opposite to him, thrust a common clay tobacco-pipe into the lad's eye, and made, apparently, a very deep wound. For several days, however, nothing was thought of the event, and but little, if any, inconvenience experienced by the boy. About the eighth or ninth day his appetite was perceived to fall off; he became lazy, feverish, and languid, and had frequent rigors; this was followed by severe pain of the head, especially of the sinciput; his tongue was much furred also. In this state he applied to a medical man, who gave him an aperient, but who was not attracted by the apparently trivial wound in the eyelid. The symptoms became more unfavorable, and he applied at the Ophthalmic Hospital, and a portion of tobacco-pipe, about two inches in length, was extracted from the orbit by Mr. J. R. Alcock. The boy was copiously bled and purged, but his sufferings continued to increase; the sight of the affected eye was lost; he became delirious; and urgent irritative fever succeeded, and it was inferred that suppuration was taking place within the cranium.

In this state the lad was sent to the Westminster Hospital on the 11th January last. He was sensible only at short intervals, and appeared to be constantly suffering the most excruciating pain; he was continually moaning and rolling his head from one side to the other, or holding it fixed in a state of apoplectic insensibility. His pulse was 140, small, irregular, feeble; bowels acting imperfectly; skin of variable temperature. He had been bled to the utmost verge of prudence, and the only means that could be judiciously employed were merely palliative. His respiration became hurried, at times occasionally laborious and stertorous, presenting nearly an apoplectic character. Blood was taken after his admission from the temporal artery, but no appreciable improvement was evident.

The affected eye and its appendages formed a large tumor, but the wound was scarcely perceptible. He died on the 12th.

*Post-mortem examination.* Much interest was felt in this case; for it was imagined the pipe must have broken the orbitar process, and penetrated the anterior lobe of the cerebrum. The corpse was examined in the presence of Mr. Guthrie, about sixteen hours after the decease of the patient. On raising the calvarium, the membranes were found a little more injected than natural, but no other morbid indication was discovered in the whole cerebral mass, save a slight opacity of the pia mater covering the pons varolii. This was found to be opposite a portion of discoloured dura mater, extending over the left cavernous sinus. An opening was made into this cavity, and a piece of pipe, an inch long, was discovered thrust between the nervus abducens and the carotid artery. Extensive disorganization, of course, prevailed throughout the whole sinus; and the existence of a foreign body of such size in such a centre of nervous sympathy, was considered not inadequate to account for the severity of the symptoms which manifested themselves. No extravasation, however, had taken place, nor any fracture of the orbitar plate. The pipe had passed under the roof of the orbit, and entered through the foramen lacerum orbitale, amongst the contents of the cavernous sinus. The carotid, though contracted a little in diameter at its point of contact with the intruded body, was yet sufficiently pervious. There was no penetration through the dura mater. There was nothing worthy of observation in any other part of the subject.

---

